# Utilizing Advanced Technologies to Augment Pharmacovigilance Systems: Challenges and Opportunities

**DOI:** 10.1007/s43441-019-00023-3

**Published:** 2019-12-28

**Authors:** David John Lewis, John Fraser McCallum

**Affiliations:** 1 Novartis Global Drug Development, Novartis Pharma GmbH, Oeflinger Strasse 44, D-79664 Wehr, Germany; 2 Department of Pharmacy, Pharmacology and Postgraduate Medicine, University of Hertfordshire, Hatfield, Hertfordshire AL10 9AB UK; 3 Product Development Safety Risk Management, Roche Products Limited, 6 Falcon Way, Shire Park, Welwyn Garden City, Hertfordshire AL7 1TW UK

**Keywords:** Pharmacovigilance, Information technology, Emerging technology, Artificial intelligence, Automation

## Abstract

There are significant challenges and opportunities in deploying and utilizing advanced information technology (IT) within pharmacovigilance (PV) systems and across the pharmaceutical industry. Various aspects of PV will benefit from automation (e.g., by improving standardization or increasing data quality). Several themes are developed, highlighting the challenges faced, exploring solutions, and assessing the potential for further research. Automation of the workflow for processing of individual case safety reports (ICSRs) is adopted as a use case. This involves a logical progression through a series of steps that when linked together comprise the complete work process required for the effective management of ICSRs. We recognize that the rapid development of new technologies will invariably outpace the regulations applicable to PV systems. Nevertheless, we believe that such systems may be improved by intelligent automation. It is incumbent on the owners of these systems to explore opportunities presented by new technologies with regulators in order to evaluate the applicability, design, deployment, performance, validation and maintenance of advanced technologies to ensure that the PV system continues to be fit for purpose. Proposed approaches to the validation of automated PV systems are presented. A series of definitions and a critical appraisal of important considerations are provided in the form of use cases. We summarize progress made and opportunities for the development of automation of future systems. The overall goal of automation is to provide high quality safety data in the correct format, in context, more quickly, and with less manual effort. This will improve the evidence available for scientific assessment and helps to inform and expedite decisions about the minimization of risks associated with medicines.

## Background

Recent technological advances in the areas of artificial intelligence and intelligent automation have profound implications for medicine and in the discovery development and post-marketing phases of the life cycle of pharmaceutical medicines. In comparison to other business sectors, the pharmaceutical industry has been relatively slow to adopt artificial intelligence and automation. As a direct consequence the industry has struggled not to fall behind in terms of the implementation of intelligent automation related to other sectors (e.g., finance). Pharmacovigilance (PV) is the science of monitoring the effects of medicinal products with the aim of identifying and evaluating potential adverse reactions [1]. Regulations and guidelines are in place to govern PV conducted by pharmaceutical companies [2–4]. PV systems are supported by secure closed relational databases. These databases are often linked to a data warehouse and advanced tools to enable the retrieval reporting and visualization of data in support of signal detection and risk management [5, 6].

## Introduction

**Figure 1.**
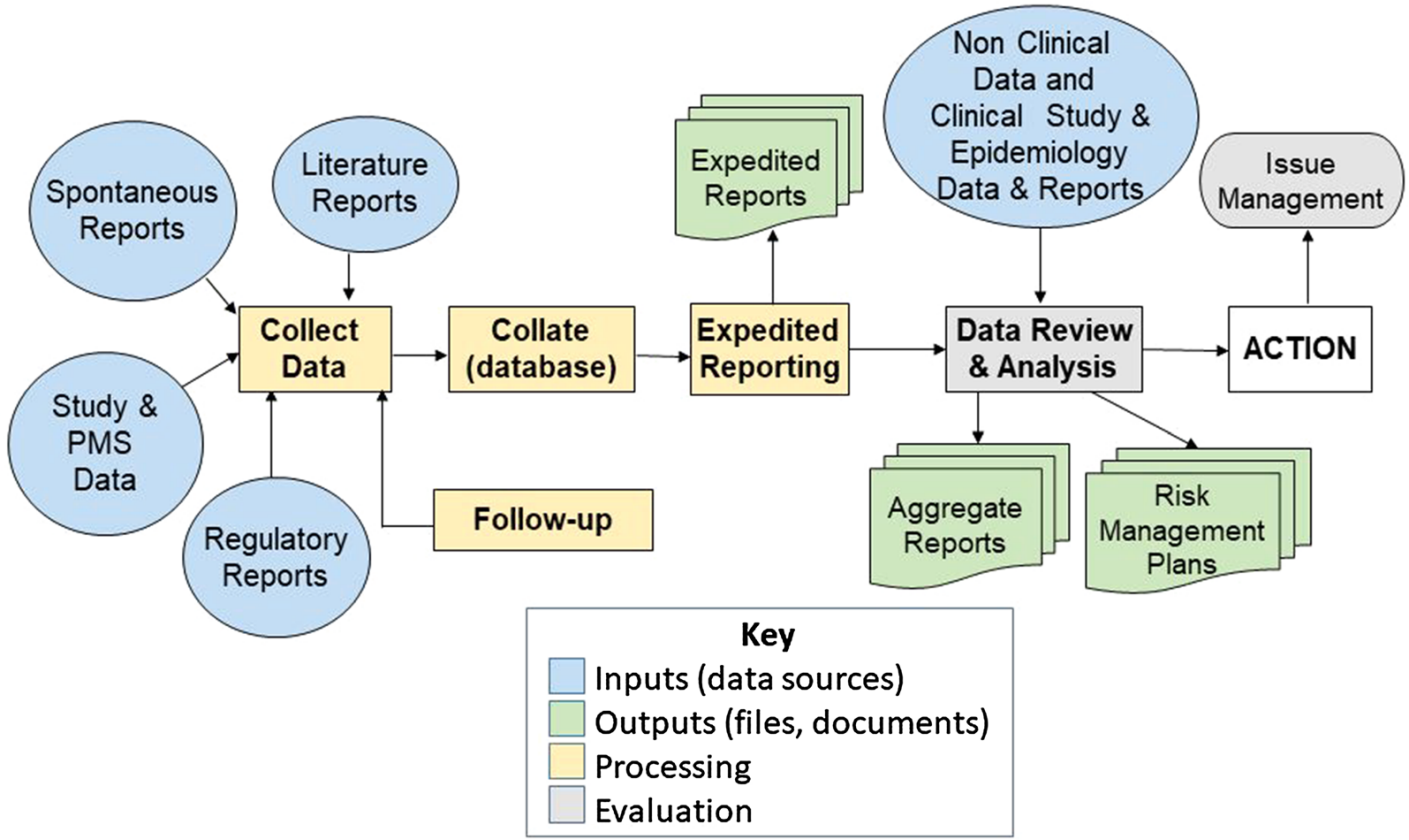
Typical Process for the Management of Individual Case Safety Reports Within a Marketing Authorization Holder.

There is a wealth of regulations concerning the obligations incumbent upon Sponsors of clinical trials [7, 8] and Marketing Authorization Holders (MAHs) for marketed medicinal products [2, 4]. These obligations mandate the collection of large and growing volumes of safety data [4], with some MAHs conducting over one million transactions involving individual case safety reports (ICSRs), medication errors, product quality complaints, and exposures to medicines during pregnancy. ICSRs comprise records of single patients from various sources (e.g., healthcare professionals, patients and carers) which are collected, collated, formatted and assessed according to a standard process [9–12]. Figure 1 shows a typical flow diagram to illustrate the processing ICSRs within a MAH.

**Figure 2.**
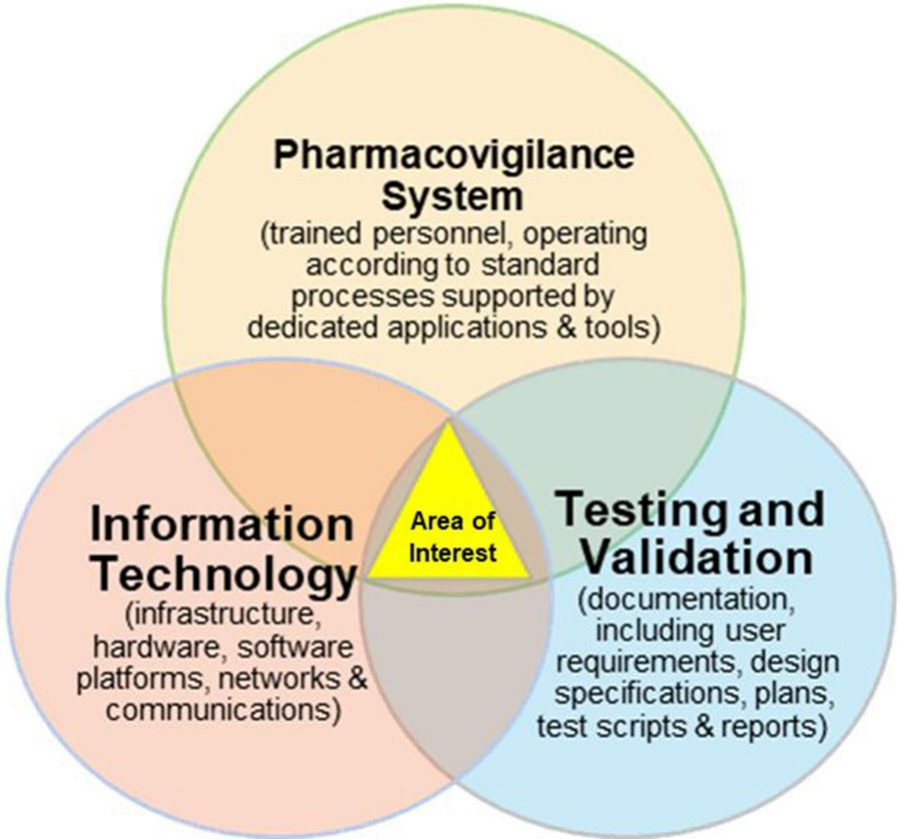
Conceptual Diagram Showing the Different Entities Relating to Automation in PV Systems.

This has challenged established business practices. It provides an opportunity to determine if, and where, new technology may add value to the traditional, paper-based PV processes. The practical implementation and deployment of advanced information technology (IT) will have implications for regulators and may affect areas involving the use of such data and an individual’s right to privacy. Figure 2 shows, at a conceptual level, these distinct areas and this paper intends to position itself at their intersection.

The TransCelerate Intelligent Automation project has been set-up to address some of the challenges [13]. Our aim is to categorize the challenges and opportunities in the context of the PV system with a view to maintain a coherent overview, using simple language to facilitate understanding. We will provide a series of considerations for the applicability for new IT, where we explain the technology and show its applicability within specific domains of the PV system (Table 2). Proposals will be made for the classification and validation of new IT and finally we will consider areas for further research.

As we progress through this paper, there are several key points to remember:

1. Significant challenges (e.g., need for large volumes of data to aid automated signal detection using disproportionality statistics) and opportunities exist (e.g., the availability of multiple methods to support disproportionality-based signal detection) when designing, deploying, and utilizing advanced IT within PV systems.
2. Rapid development of IT will outpace the regulations applicable to PV systems; validation of advanced technologies is essential to ensure that these systems remain fit for purpose (e.g., scalable to future standardized knowledge ontologies and source data) [12]
3. Automation of PV processes can provide high quality safety data in the correct format, in context, more quickly, and with less manual effort, thereby improving the evidence available for timely scientific assessment.

## Pharmacovigilance Systems Operated by Marketing Authorization Holders

The PV system within a MAH is an important contributor to the evidence base required for the approval of a licensed medicine. Safety, quality, and efficacy must be established in order for a product license to be granted. PV systems operated by MAHs must be managed according to Good Pharmacovigilance Practice standards and, under applicable law, must include at least the following capabilities: [2, 3, 14, 15]

1. To perform ongoing benefit−risk assessment throughout the life cycle of the medicinal product
2. To collect, collate, and manage case reports of adverse reactions (including overdose, misuse, abuse, addiction, or tolerance), reports of exposures to medicines during pregnancy, reports of medication errors, and suspected counterfeit or substandard medicinal products
3. To report both ICSRs and in aggregate [periodically according to legal obligations to the Health Authorities and other stakeholders within countries and regions (e.g., healthcare professionals, ethics committees, patients, carers, etc.)]
4. To conduct safety monitoring within clinical trials according to Good Clinical Practice standards, ensuring that important safety information is documented and reported
5. To design, implement, and conduct post-authorization safety studies (PASS) to investigate, identify, characterize, and/or quantify a safety hazard; to confirm the safety profile of a medicine; and/or to measure the effectiveness of risk-management measures
6. To identify safety signals causally associated with medicinal products throughout clinical development and in the post-marketing phase
7. To prepare a Risk Management Plan and/or Risk Evaluation and Mitigation Strategy with additional Risk Minimization Materials where required
8. To monitor the effectiveness of risk minimization measures and to adjust or amend materials to reduce risks to patients
9. To identify if there are product quality problems in medicines resulting in adverse drug reactions (ADRs) and, more generally, support the identification of medicine quality issues
10. To apply resulting information from pharmacovigilance for the benefit of public health programs, individual patients, and national medicines policies and treatment guidelines
11. To develop and maintain drug utilization information
12. To identify issues associated with unregulated prescribing and dispensing of medicines

The WHO also sets the expectation that a PV system must enable the provision of effective communication on aspects related to medicine safety, including dispelling unfounded rumors of toxicity attributed to medicines and/or vaccines [16, 17].

## Current Uses of Information Technology in Pharmacovigilance: Rule-Based Static Systems

Current PV systems rely on binary-logic based algorithms to aid safety data management. Some examples of programmatic algorithms in rule-based static systems are provided in Table 1. These user-managed rules apply consistent, objective expert knowledge in a standardized manner; the results of algorithmic data processing are therefore known and understood. This approach takes best performance and converts it into the standard process. Validation of these rules follows traditional methods, such as script-based user acceptance testing (UAT). Five (5) PV system domains are defined in the European Good Pharmacovigilance Practice Annex 1 [1] and briefly described here:

**Table 1.** Algorithmic Functions in Rule-based Static Systems.

Algorithm Purpose	Use Case
Association	Quality Management System (QMS): Detection of data outliers in clinical trials or post-marketing studies (e.g., abnormal hematology results indicative of a blood dyscrasia, changes of heart rate or rhythm, which may represent an adverse reaction to medical treatment) Detection of patients with undiagnosed Gilbert’s syndrome (by careful assessment of liver function test results and evaluation of any associated signs or symptoms using single-patient profiles) ICSRs: Duplicate checking of ICSRs (identification of potential duplicate reports—for example, the same patient reported by the attending doctor and the pharmacist that dispensed the medicinal product by identifying links between selected data fields such as age, gender, start and stop dates of medical treatment, adverse reaction terms, outcome, etc.)
Filtering	Signal management: Prioritization of medical review of adverse event terms using statistical disproportionality scores which exceed a pre-determined threshold level (counts above the threshold represent an excess of observations of a particular adverse event versus the expected value) ICSRs: Processing and management of ICSRs, e.g., workflow with prescribed routing of ICSRs for processing (see Introduction and Fig. 1 for details)
Prioritization	Risk management: Identification of potential risks (an untoward occurrence for which there is some basis for suspicion of an association with the medicinal product of interest but where this association has not been confirmed) versus identified risks (an untoward occurrence for which there is adequate evidence of an association with the medicinal product of interest) [1]
Classification	Aggregate reporting: Categorizing ICSRs within a Periodic Safety Update Report (PSUR), e.g., serious versus non-serious and reported by healthcare professional versus reported by a patient

- **Quality (management) system** (**QMS**) **for the PV system:** The organizational structure, responsibilities, procedures, processes, and resources of the PV system as well as appropriate resource management, compliance management, and record management. The QMS is part of the PV system [18].
- **Risk management system:** A set of PV activities and interventions designed to identify, characterize, prevent, or minimize risks relating to a medicinal product, including the assessment of the effectiveness of those activities and interventions [19].
- **Management of ICSRs:** Collection, collation, processing, and assessment of safety data to standardize the format and content of a relational database used for the reporting of one or several suspected adverse reactions to a medicinal product that occur in a single patient at a specific point of time [20].
- **Aggregate reporting:** Periodic reports summarizing safety data received over a fixed-term for a specified medicinal product, sometimes including a detailed comparison with the cumulative safety data for the product. Examples include the Periodic Benefit Risk Evaluation Report (PBRER) [21].
- **Signal management:** A set of activities performed to determine whether, based on an examination of ICSRs, aggregated data from active surveillance systems or studies, scientific literature information or other data sources, there are new risks associated with an active substance or a medicinal product or whether known risks have changed, as well as any related recommendations, decisions, communications, and tracking [22].

### Artificial Intelligence (AI)-Informed Static Systems

Artificial Intelligence (AI) is an all-embracing term for the simulation of human intelligence processes by computer systems. AI encompasses a wide range of technologies including following rules, reasoning (using rules to reach approximate or definite conclusions), learning, and self-correction. These technologies can be seen in a number of PV system domains including ICSRs [20], aggregate reporting, risk management, signal management, and the QMS supporting the PV system. The arguments in favor of the utility of AI that make this technology applicable to multiple domains within pharmacovigilance systems are based initially, at least, on the elimination of human error, standardization of processes, expediting processing cycle times, and reducing manual effort. A trend towards adoption of AI-informed processing is evident where the results are largely known and understood. Validation is based on the correlation of the human-processed data with the results from the AI-informed process; a use case could be use of machine learning to code terms or products where such terms are ambiguous, have errors, abbreviations, or use slang terms.

### Putative Adoption of Dynamic Systems with Binary or Retrospective Outcome or Predictive Outcome

Dynamic systems can be integrated into PV systems and produce binary (or retrospective) outcomes in which the results make sense to a human and the method is understood by a human. Dynamic systems can also be used to produce predictive outcomes in which the results make sense to a human and the method can be comprehended by a human. PV systems are invariably dynamic; for this reason, implementation and validation is problematic. At this time, there is no clear regulatory guidance, nor is there a consensus of approach concerning the adoption of automation in this domain. One use case would be intelligent workflow for the processing of ICSRs. In this setting, a company could employ automation to direct designated staff to conduct specific tasks and which may be semi-automated. This would facilitate processing by helping to balance in real time the amount and priority of the work to the availability and skill level of the people supporting the process, leading to improvements in quality and compliance of the output.

### Future Pharmacovigilance Systems: Adoption of Emerging Technologies

Consideration of emerging technologies will be on a case-by-case basis. While it is speculative to pre-determine the applicability of such technologies to PV systems, we are aware of some examples of pilot testing.

Emerging technologies facilitating innovation in PV are many and complex. While the IT professionals responsible for creating these technologies understand their mechanics, it is not always clear to the professionals attempting to bring these to bear on the science and process of PV. We describe some IT emerging technologies and how they can be used in PV in Table 2. Lengthy and detailed technical definitions are available elsewhere [23, 24].

**Table 2.** Intelligent Automation Technologies and Potential Applicability to PV.

Term	Description	PV System Domain
Artificial intelligence (AI) [25]	An all-embracing term for the simulation of human intelligence processes by computer systems. AI encompasses a wide range of technologies including following rules, reasoning (using rules to reach approximate or definite conclusions), learning, and self–correction	ICSR [26], Aggregate, risk management, signal management, QMS
	The arguments in favor of the utility of AI that make this technology applicable to multiple domains within pharmacovigilance systems are based initially, at least, on the elimination of human error and standardization of processes	
Cognitive computing	The simulation of human reasoning in a computer system and often synonymous with AI. The goal of cognitive computing is to create automated IT systems that are capable of solving problems with little or no human assistance using machine–learning techniques	ICSR, aggregate [27], signal management [27], risk management [27], QMS
Machine learning (ML) [26]	An application of AI that provides computer systems with the ability to automatically learn and improve from experience without being explicitly programed. Machine learning focuses on the development of computer programs that can access data and use it to learn for themselves and adapt over time, e.g., applying historic understanding to predict accurate outcomes from current inputs. Deep learning is distinct from machine learning largely by depth of the neural network or the number of layers of the neural network. There are several methods used to train the machine, based upon the task under consideration (e.g., classification, clustering, association, etc.). Supervised learning has been tested in PV, largely in ICSR processing, where a human–annotated answer file (“ground truth”) is used to teach the machine learning algorithm(s) [28, 29]. Unsupervised learning and reinforcement learning methods, where there is no “ground truth”, may have utility in signal management, as they would avoid introduction of bias in identifying potential signals	ICSR [29, 30], aggregate, signal management, risk management [31], QMS
Neural network	A computer system modeled on the neuronal structure of the mammalian brain. Neural networks are typically organized in layers made up of a number of thousands of interconnected nodes. Data are presented to the network via an input layer which communicates to one or more hidden layers where the actual processing is done. These hidden layers then link to an output layer where the answer is surfaced. Examples include convolutional neural networks and recurrent neural networks	ICSR, aggregate, signal management [32], risk management, QMS
Semantic search	Semantic searching seeks to improve search accuracy by understanding the searcher’s intent and the contextual meaning of terms to generate relevant results	ICSR [33], aggregate, risk management, signal management
Blockchain	A blockchain is a continuously growing list of records, called blocks, which are linked and secured using cryptography. By design, a blockchain is resistant to modification of the data; also, it records transactions between two parties efficiently and in a verifiable and permanent way	ICSR, aggregate, risk management, signal management, QMS
Arguments and use case for blockchain	Blockchain technology has been widely adopted in financial systems and is used for tracking, tracing, auditing, and monitoring transactions. We are aware of potential applicability in healthcare and biomedical research [34]. Fourth generation blockchain could be used to operate and monitor a co–licensing contractual agreement between two different legal entities in which all transactional data are managed within the blockchain architecture. The implication is that all aspects of the partnership affecting the PV system are contained within a single protected environment, traceable, and readily available for audit or inspection. It remains unclear how such an application might be validated	
Optical character recognition (OCR)	OCR recognizes characters within a digital image. It is commonly used to recognize text in scanned documents. While OCR was designed for printed text, it can be used to verify handwritten text	ICSR, QMS
Natural language processing (NLP)	NLP helps computers understand human language, aiding interactions with humans in their own language and scaling language-related tasks. NLP can extract text from unstructured sources, interpret it, determine sentiment, and understand importance to create meaning. We are also aware of the use of word embeddings (i.e., representation of words as vectors) [38]	ICSR [35–37], aggregate, signal management
Machine translation (MT)	The application of computers to the task of translating texts from one natural language to another	ICSR, aggregate, risk management
Speech recognition (speech-to-text)	Use of ML technologies to enable the recognition of spoken language and conversion of this into text	ICSR
Speech synthesis (text-to-speech)	The use of computer systems to produce artificial human speech which is understandable to humans in natural language	ICSR, signal management, risk management
Arguments and use case for the above text and language technologies	Variously combined, OCR, NLP, and MT technologies have the potential to simplify and standardize the intake of ICSR-containing data into the PV system. It is less clear how speech synthesis or recognition could be integrated; however, conceptually, these technologies could be employed to gather safety data from patients or prescribers with real-time querying to improve completeness of initial data capture and reduce follow-up burden	
Machine vision computer vision	The ability of a computer to mimic sight and recognize objects to enable decisions or additional processing. Examples may include OCR and/or the interpretation of diagnostic test results	ICSR
Natural language generation (NLG)	A computer process that automatically transforms structured data into a written or unstructured narrative. In order for any NLG software to produce human-ready narrative, the format of the content must be outlined (through templates, rules-based workflows, and intent-driven approaches) and then fed structured data from which the output is created	ICSR, aggregate, risk management, signal management
Autonomous software	A software entity that carries out operations on behalf of a user with a degree of independence, employing some knowledge or representation of the user’s goals or desires	ICSR, aggregate, signal management, risk management, QMS
Robotic process automation (RPA) [39]	RPA utilizes software (“virtual workers” or “bots”) to perform traditionally manual activities comprising high-volume, repetitive, rule-based processes involving structured data. RPA mimics execution of the repetitive activities without intervention or assistance	ICSR, aggregate, signal management, QMS
Desktop automation	Desktop automation is automation within a computer desktop to provide assistance or guidance to a human resource upon demand. It can perform activities such as copying and pasting information, data entry, and opening applications. These activities occur on an employee’s desktop and can be initiated by one or a combination of steps, such as a button click or switching tabs	ICSR, aggregate, signal management
Bots [39]	Bots are programs which carry out RPA. Bots work 24/7, at machine speed, without pausing, and are fully compliant with the process. Changes can be implemented instantly without training. Bots are scalable to suit the process. A variation is a smartbot, which is enriched by AI	ICSR [39], aggregate, signal management, risk management, QMS
Chatbots [40]	Bots which conduct a conversation via audio or text methods and designed to convincingly simulate human conversation. Some chatbots are simple in operation, while others use NLP	ICSR [40], Signal management, risk management, QMS [40]
Arguments and use case for the above automation technologies	Automation technologies, while varied, are easily integrated to operate standardized workflows, which are currently heavily human-orientated. Orchestrated design of these workflows, combined with other advanced technologies, have the ability to mitigate manual, error-prone, and repetitive administrative tasks in ICSR management	
Image recognition	The use of cameras, machine vision, and AI to enable a computer system to identify objects, places, people, and writing in static and video images	ICSR [41]
Text analytics Text mining	The examination of large collections of written resources to generate new evidence or insight. Using OCR and NLP, the goal of text mining is to discover relevant information in unstructured text, transforming or structuring this into data that can be used for further analysis or processes, e.g., ingesting an email directly into specific database fields or collation of relevant information in a clinical study report into an aggregate report	ICSR [42], aggregate, signal management [43]
Sentiment analysis	The contextual identification and extraction of meaning from text. It utilizes deep learning to understand intentions and reactions and determine if an expressed opinion is favorable, unfavorable, or neutral, and to what degree. An example could be the assignment of reporter causality assessment relating to an adverse event for an administered medicinal product	ICSR [44]
Advanced analytics	The automated or semi-automated analysis of data using sophisticated tools such as machine learning, neural networks, and data mining to discover deeper insights, make predictions, or generate recommendations beyond those of traditional business intelligence	Aggregate, signal management, risk management [31], QMS
Predictive analytics and predictive reasoning	This specific branch of advanced analytics utilizes current and historical data to draw inferences to forecast activity, behavior, and trends. It involves applying statistical analysis techniques, analytical queries, and ML to data sets to create predictive models of a particular event happening	Signal management [27, 31], risk management [27, 31]
Arguments and use case for the above analytic technologies	Use of analytic technology may be able to reduce the burden on human resources to isolate safety-relevant information from large documents, such as clinical or pre-clinical study reports, including those from outside sources. This would allow safety personnel to focus on the impact to patient safety (signal management, aggregate reporting) and reduce the administrative burden of managing complex documents	

## Ethical and Policy Implications of Automation Within PV Systems

We recognize that regulations are in place concerning the processing of personal data. The science of PV relies on the consistent and diligent application of high-quality data management techniques which can be improved by automation. The primary purpose of PV is to reduce or minimize risks, which in turn contributes to optimizing the use of medicinal products. In general, safety evaluation is agnostic of personal data; hence, there is a limited likelihood of any invasion of privacy or risk of breach of confidentiality. Nevertheless, the use of automation in PV systems must be very carefully assessed to ensure compliance with data privacy regulations [45, 46]. As an obligation related to the QMS for the PV system, a company would need to consider the degree of human oversight necessary to provide a trusted, reliable, consistent, and valid output.

Beyond the technological considerations, MAHs will need to consider the procedural, organizational, and capability changes required to implement more advanced intelligent automation solutions. Novel technologies provide significant opportunities to enhance the conduct of PV:

- Novel methodologies to demonstrate the oversight of capability of the PV system to protect the wellbeing of patients
- Opportunity to question underlying assumptions and potentially eliminate traditional process steps, such as triage, to improve overall quality and performance of the PV system
- Re-examine resourcing in PV: Implementation of intelligent automation technologies is in itself a sourcing strategy and, with sufficient scale, provides an opportunity to elevate quality, efficiency, capacity, and performance in PV

Beyond validation of new IT, there are also other considerations that will be critical to its implementation:

- The right of all individuals to privacy and to protection of personal data concerning health matters. Regulations exist at both regional and national levels in order to safeguard the privacy of safety data from which an individual may be identified [45, 46]. The European General Data Protection Regulation (GDPR), for example, applies to organizations that control or process personal data of European Union (EU) residents whether or not they have a physical presence within the EU.
- Skills and competencies of individuals working within the PV system. Currently these are built around established methods but use of machine learning (for example) will lead to the disruption of the traditional PV and IT interfaces [26, 27, 40, 42]. Already, there is evidence that AI can equal or exceed human performance in various areas of medical science [47–51].
- Change management with impacted functions and individuals [52]. The PV system spans many areas beyond the safety department of the MAH, and the use of new IT will cause impact to many upstream and downstream stakeholders. It is likely that such implementation will result in unforeseen challenges but produce equally unforeseen opportunities to improve the entire PV system.
- Impact of intelligent automation on Business Continuity planning. With increasing levels of automation, it may be prudent to focus efforts on resumption of automated activities and move away from manual workarounds. However, it will be important to maintain flexibility to comply with the regulations surrounding PV and continue vigilance activities during an interruption to protect patient safety.
- Preparation, conduct, and completion of corrective actions and preventative actions (CAPAs) resulting from audit and inspection. Certain aspects of intelligent automation are not human intelligible. It is vital in audit and inspection to provide justifiable evidence that the automation deployed is fit for purpose, and that the MAH has demonstrable oversight of their PV system regardless of the level and method of automation employed within it.

## Discussion

We have introduced some of the emerging IT landscape as applicable to PV systems and introduced elements of intelligent automation that would promote continuous improvement of established PV systems. Intelligent automation has the potential to reduce cycle times, improve quality, create efficiencies, and reduce costs, all of which lead to better allocation of resources and can improve patient outcomes. This, in turn, contributes to the improvement of public health. We recognize that it is incumbent upon MAHs to take a responsible and considered approach to the use of automation in PV systems. It is not possible for regulatory agencies to legislate for advanced IT that evolves at a much faster rate than regulations can be published. Each of the technologies identified in this paper has the potential to yield efficiencies and improvements of quality not only for ICSRs, but also in the context of aggregate reporting, signal detection, risk management, and the QMS for the PV system.

The future is impossible to predict; however, it can be envisaged that at some point, if all stakeholders operate within a single global database, then all activities could be run under blockchain technologies. This may obviate any need for audits or inspections, as all interactions would be subject to tracking and scrutiny.

## Conclusions

We recognize that the rapid development of new technologies will invariably outpace the regulations applicable to PV systems. Nevertheless, we believe that PV systems may be improved by intelligent automation. It is incumbent on the owners of PV systems to oversee applicability, design, deployment, performance, validation, and maintenance of advanced technologies to ensure that the PV system continues to be fit for purpose. It is equally, if not more, important that intelligent automated PV systems should be trusted. Our aim is to provide the right type of safety data in the right format and context, thereby increasing the quality of the evidence available for scientific evaluation to inform decisions optimally. Ultimately, our views concerning PV systems are similar to those expressed by other authors in the field of biomedical sciences [53–55]. We believe that PV system owners will have to adapt to artificial intelligence by the progressive, intelligent adoption of new technologies, commencing with process automation. It has already been established that algorithmic processing can outperform humans when performing binary, repetitive tasks [56]. The question that we cannot yet answer, is exactly how far data-driven, high-performance PV can be taken by intelligent automation? Further, the evolution of PV in recent years along with the increasing volume of generated and gathered data point to the importance of adopting more advanced technologies that will not only automate but also improve the overall process. If we paraphrase Turing’s original hypothesis from 19 [50, 57] then, provided we establish what are proven facts in PV systems, then the conjectures that may result from the intelligent use of AI are of potentially great importance because they suggest useful lines of research.
